# Neuroprotection by Caffeine in Hyperoxia-Induced Neonatal Brain Injury

**DOI:** 10.3390/ijms18010187

**Published:** 2017-01-18

**Authors:** Stefanie Endesfelder, Ulrike Weichelt, Evelyn Strauß, Anja Schlör, Marco Sifringer, Till Scheuer, Christoph Bührer, Thomas Schmitz

**Affiliations:** 1 Department of Neonatology, Charité, Universitätsmedizin Berlin, 13353 Berlin, Germany; evelyn.strauss@charite.de (E.S.); till.scheuer@charite.de (T.S.); christoph.buehrer@charite.de (C.B.); thomas.schmitz@charite.de (T.S.); 2 Department of Physiology, Charité, Universitätsmedizin Berlin, 10117 Berlin, Germany; ulrike.weichelt@charite.de; 3 Department of Biochemistry and Biology, University of Potsdam, 14476 Potsdam, Germany; anja.schloer@uni-potsdam.de; 4 Department of Anesthesiology and Intensive Care Medicine, Charité, Universitätsmedizin Berlin, 13353 Berlin, Germany; marco.sifringer@charite.de

**Keywords:** anti-oxidative response, caffeine, hyperoxia, oxidative stress, preterm infants, developing brain

## Abstract

Sequelae of prematurity triggered by oxidative stress and free radical-mediated tissue damage have coined the term “oxygen radical disease of prematurity”. Caffeine, a potent free radical scavenger and adenosine receptor antagonist, reduces rates of brain damage in preterm infants. In the present study, we investigated the effects of caffeine on oxidative stress markers, anti-oxidative response, inflammation, redox-sensitive transcription factors, apoptosis, and extracellular matrix following the induction of hyperoxia in neonatal rats. The brain of a rat pups at postnatal Day 6 (P6) corresponds to that of a human fetal brain at 28–32 weeks gestation and the neonatal rat is an ideal model in which to investigate effects of oxidative stress and neuroprotection of caffeine on the developing brain. Six-day-old Wistar rats were pre-treated with caffeine and exposed to 80% oxygen for 24 and 48 h. Caffeine reduced oxidative stress marker (heme oxygenase-1, lipid peroxidation, hydrogen peroxide, and glutamate-cysteine ligase catalytic subunit (GCLC)), promoted anti-oxidative response (superoxide dismutase, peroxiredoxin 1, and sulfiredoxin 1), down-regulated pro-inflammatory cytokines, modulated redox-sensitive transcription factor expression (Nrf2/Keap1, and NFκB), reduced pro-apoptotic effectors (poly (ADP-ribose) polymerase-1 (PARP-1), apoptosis inducing factor (AIF), and caspase-3), and diminished extracellular matrix degeneration (matrix metalloproteinases (MMP) 2, and inhibitor of metalloproteinase (TIMP) 1/2). Our study affirms that caffeine is a pleiotropic neuroprotective drug in the developing brain due to its anti-oxidant, anti-inflammatory, and anti-apoptotic properties.

## 1. Introduction

Advances in neonatal intensive care have led to a significant increase in the survival rate of premature infants, but extremely premature infants have a higher risk of dying or suffering permanent and serious damage [1,2]. Up to 50% of surviving extremely preterm infants show cognitive deficits or behavioral problems during the later stages of development [3]. The sequelae of prematurity are described to be triggered by oxidative stress and free radical-mediated cell and tissue damage, leading to the term “oxygen radical disease of the prematurity” [4,5,6].

There are several reasons for the high susceptibility of preterm infants to oxidative damage: (i) birth is associated with a dramatic change of intrauterine hypoxic milieu to a relatively hyperoxic extrauterine environment, and this relative hyperoxia can be enhanced by supplemental oxygen [7,8]; (ii) premature infants are less able to cope with the oxygen-rich environment of extrauterine life because their antioxidant defense system is poorly developed [9]; and (iii) preterm infants have increased susceptibility to infections [5].

Oxidative stress can be defined as an imbalance between the amount of reactive oxygen species (ROS) and the intracellular and extracellular antioxidant protection systems. The antioxidative defense system undergoes developmental changes during the neonatal period, resulting in a relevantly lower intracellular defense in preterm infants compared to term infants [10].

In addition to the understanding of the pathology of oxidative stress and the associated effects on the development of premature infants, additional strategies must be developed. Recent studies have proposed that caffeine presents antioxidant activity and therefore, protects human against disorders associated with oxidative stress [11,12]. The methylxanthine caffeine is used as a first-line pharmacotherapy against apnoea in preterm infants [13]. Caffeine has a higher therapeutic index and a longer half-life compared to other methylxanthines. In addition, to the reduction of the frequency of apnoea, caffeine has additional short- and long-term effects [14,15]. As an adenosine receptor antagonist caffeine improves neonatal outcome, shows neuroprotective effects in the developing brain [16,17], has anti-inflammatory effects [18,19], decreases rates of bronchopulmonary dysplasia (BPD) and death [14,20], and shortens the duration of mechanical ventilation [14,21]. Side effects of caffeine have been described [22,23] to include tachycardia, higher oxygen consumption, and transient decrease of the growth rate in very low birth weight infants [24].

Up to date, it is not yet clarified whether caffeine can also act as a free radical scavenger. Shi et al. reported that caffeine may act as an antioxidant scavenger, thus explaining the observed anticarcinogenic properties of caffeine and related methylxanthine compounds [25]. Furthermore, caffeine prevented lipid peroxidation, reduced oxidative DNA damage [26], modulated oxidative stress in rat liver [27], and showed immunmodulatory effects under oxidative stress in the neonatal rat brain [16] and immune cells [28]. Due to the anti-oxidant properties per se and/or by the anti-inflammatory and anti-apoptotic effects of caffeine [16,29,30], which seem to be adenosine receptor-mediated [30,31], caffeine would be a promising pleiotropic drug. Therefore, the aim of this in vivo study in a neonatal oxidative stress model was to investigate how caffeine affected the immature rodent brain against high oxygen exposure.

## 2. Results

### 2.1. Hyperoxia Induces Oxidative Stress Which Is Counteracted by Caffeine

**Figure 1:**
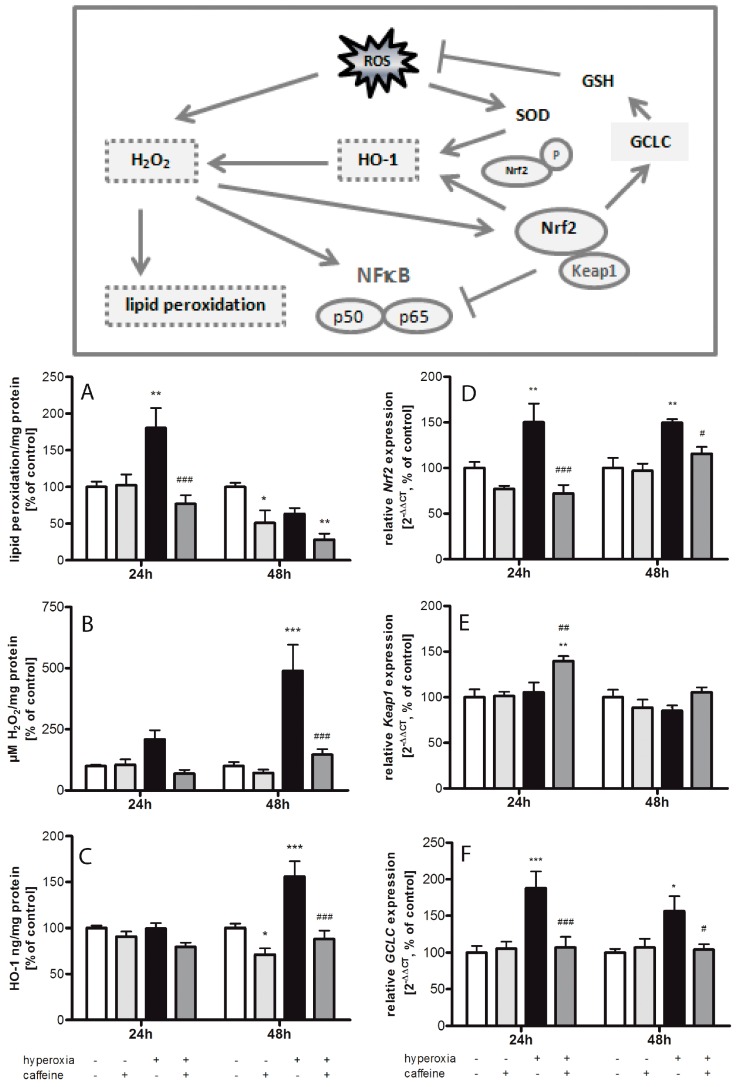
Caffeine reduces oxidative stress responses during exposure to hyperoxia. (**Box**) Reactive oxygen species imply hydrogen peroxide, which promotes lipid peroxidation. The antioxidant enzyme response is induced, which leads inter alia to an activation of heme oxigenase-1 (HO-1). The Nrf2-Keap1 system plays alongside the NFκB pathway an essential role in the implementation of the antioxidant gene regulation in response to oxidative stress. The catalytic subunit of glutamate-cysteine ligase (GCLC) is upregulated by Nrf2. Quantitation of brain homogenates by ELISA of: (**A**) TBARS/lipid peroxidation; (**B**) H_2_O_2_; and (**C**) HO-1; and mRNA expression by quantitative real-time PCR of: (**D**) *Nrf2*; (**E**) *Keap1*; and (**F**) *GCLC*. Groups are shown as normoxia (**white bars**), hyperoxia (**black bars**), with and without caffeine (**dark grey** and **light grey bars**, respectively) as mean ± SEM, *n* = 4–5 per group per time point. The 100% value is: (**A**) 1.268 and 1.959 µM/mg protein; (**B**) 0.887 and 2.946 µM/mg protein; (**C**) 3.604 and 2.891 ng/mg protein; (**D**) 1.008 and 1.029 *C*_T_; (**E**) 1.014 and 1.029 *C*_T_; and (**F**) 1.016 and 1.015 *C*_T_ for 24 and 48 h groups, respectively. Data were analyzed by two-way ANOVA with Bonferroni post hoc test, with * *p* < 0.05, ** *p* < 0.01, and *** *p* < 0.001 versus control (atmospheric air), and ^#^ *p* < 0.05, ^##^ *p* < 0.01, and ^###^ *p* < 0.001 versus hyperoxia (80% oxygen without caffeine).

Thiobarbituric acid reactive substances (TBARS) were increased in brain tissue of newborn rats exposed to 24 h of hyperoxia to 180% ± 27.4% (*p* < 0.01) compared to litter control mates kept in atmospheric air (Figure 1A). This was reduced by a single dose of caffeine (77% ± 11.8%; *p* < 0.001). In control animals, caffeine did not affect TBARS levels. Changes in TBARS after 48 h hyperoxia were not significant in comparison to matched normoxic litters. Interestingly, there was also a significant decrease in TBARS in normoxic newborn rats 48 h after the single administration of caffeine (51% ± 17.2%; *p* < 0.05).

To confirm the induction of oxidative stress by exposure to hyperoxia, we analyzed hydrogen peroxide concentrations through enzyme linked immunosorbent assay (ELISA) measurement in neonatal rodent brains after hyperoxia and normoxia with and without caffeine treatment (Figure 1B). Hyperoxia leads to a highly significant increase of hydrogen peroxide after 48 h exposure duration (489% ± 106.7%; *p* < 0.001) which was blocked by caffeine (147% ± 22.6%; *p* < 0.001). Treatment with caffeine in animals kept under normoxic conditions had no effect on hydrogen peroxide concentrations.

Heme oxigenase-1 (HO-1) protein levels were markedly increased in brain tissue after 48 h of hyperoxic stimulation (156% ± 16.8%; *p* < 0.001) compared with brain tissue from rats under normoxia (Figure 1C). Caffeine reduced the protein level significantly after 48 h hyperoxia (88% ± 8.9%; *p* < 0.001). HO-1 is not increased after 24 h hyperoxia. However, caffeine treatment reduced HO-1 levels after 48 h in control litters kept in room air (71% ± 15.6%; *p* < 0.05).

NFE2-related factor 2 (*Nrf2*) (Figure 1D) was significantly induced both after 24 h (150% ± 20.3%; *p* < 0.01) and after 48 h (150% ± 3.9%; *p* < 0.01) of hyperoxia, which was blocked by caffeine (24 h to 72% ± 9.2%; *p* < 0.001, and 48 h to 116% ± 7.3%; *p* < 0.05). Contrary to *Nrf2*, gene expression of Kelch-like ECH-associated protein 1 (*Keap1*; Figure 1E) was not affected by hyperoxia alone, but was significantly elevated by caffeine after 24 h exposure to hyperoxia (140% ± 5.3%; *p* < 0.01). Caffeine did not influence the expression of *Keap1* at atmospheric air.

The glutamate-cysteine ligase (GCL) consists of two separate coded subunits, a catalytic (GCLC) and a modifier (GCLM), which catalyzes the rate-limiting phase of cellular antioxidant glutathione (GSH). *GCLC* (Figure 1F) mRNA expression was significantly induced both after 24 h (188% ± 23.0%; *p* < 0.001) and after 48 h (156% ± 20.4%; *p* < 0.05) of hyperoxia, which were significantly reduced by caffeine (24 h to 107% ± 14.4%; *p* < 0.001, and 48 h to 104% ± 7.8%; *p* < 0.05). Single treatment with caffeine in animals kept under normoxic conditions had no effect on *GCLC* expression.

### 2.2. Regulating Effect of Caffeine on the Imbalance of the Sulfiredoxin/Peroxiredoxin System after Hyperoxia

**Figure 2:**
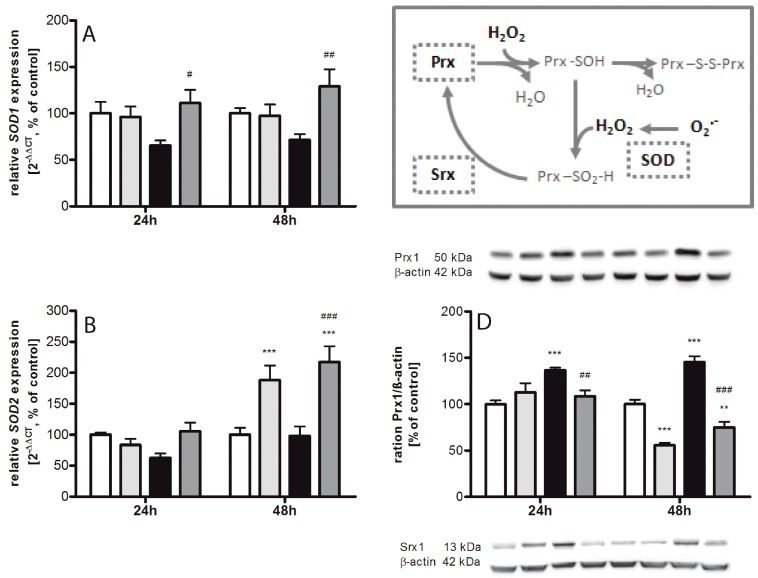

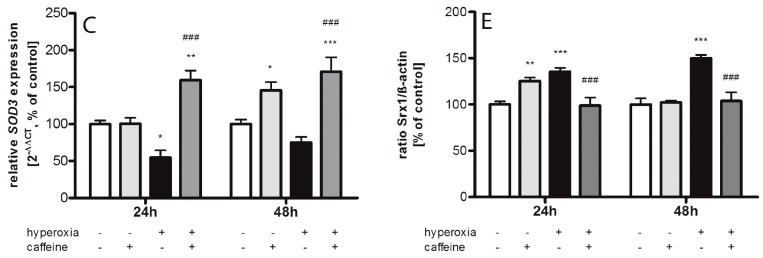
Caffeine modulates antioxidative enzymes. (**Box**) The high production of ROS under oxidative stress requires the existence of a set of ROS scavenger mechanisms. These are firstly, the group of superoxide dismutase (SOD), which are not only able to scavenge ROS but also repair cell damage and possibly serve as redox sensors, and secondly, the thiol-based antioxidants peroxiredoxin (Prx) as well as sulfiredoxin (Srx), which are major internal housekeeping antioxidant molecules that act as redox switches to modulate homeostasis. SOD isoform mRNA expression was analyzed in brain homogenates. Quantification of mRNA expression by quantitative real-time PCR of: (**A**) *SOD1*; (**B**) *SOD2*; and (**C**) *SOD3*; and protein expression measured by Western blot of: (**D**) Prx1; and (**E**) Srx1. Groups are shown as normoxia (**white bars**), hyperoxia (**black bars**), with and without caffeine (dark grey and light grey bars, respectively) as mean ± SEM, *n* = 4–5 per group per time point. The 100% value is: (**A**) 1.031 and 1.006 *C*_T_; (**B**) 1.002 and 1.025 *C*_T_; (**C**) 1.005 and 1.007 *C*_T_; (**D**) 0.87 and 0.61 ratio intensity/mm^2^; and (**E**) 0.39 and 0.67 ratio intensity/mm^2^, for 24 h and 48 h groups, respectively. Data were analyzed by two-way ANOVA with Bonferroni post hoc test, with * *p* < 0.05, ** *p* < 0.01, and *** *p* < 0.001 versus control (atmospheric air), and ^#^ *p* < 0.05, ^##^ *p* < 0.01, and ^###^ *p* < 0.001 versus hyperoxia (80% oxygen without caffeine).

Acute exposure to high oxygen leads to exhaustion of superoxide dismutases 3 (*SOD3*). *SOD1* (Figure 2A) does reveal not statistically significant differences (at 24 h 66% ± 5.4% and at 48 h 72% ± 6.1%), *SOD2* (Figure 2B) to 63% ± 7.2% at 24 h, but *SOD3* (Figure 2C) was significantly reduced to 55% ± 9.6% (*p* < 0.05) at 24 h. In comparison to hyperoxia without caffeine, we found significant increases for *SOD1* to 111% ± 14.4% (*p* < 0.05) and 129% ± 18.5% (*p* < 0.01), for *SOD3* to 159% ± 13.1% (*p* < 0.001) and 171% ± 19.3% (*p* < 0.001) at 24 h and 48 h, respectively, and for *SOD2* to 217% ± 25.5% (*p* < 0.001) at 48 h. Under 48 h normoxic conditions, a single administration of caffeine leads to an increase of *SOD2* (188% ± 23.3%, *p* < 0.001) and *SOD3* (146% ± 11.2%, *p* < 0.05). Correspondingly, increasing peroxiredoxin (Prx) 1 and sulfiredoxin (Srx) 1 expression were observed at both time points. Prx1 protein expression (Figure 2D) was increased to 137% ± 2.8% (*p* < 0.001) at 24 h and to 145% ± 6.2% (*p* < 0.001) at 48 h, and Srx1 protein expression (Figure 2E) to 135% ± 4.3% (*p* < 0.001) and 150% ± 9.3% (*p* < 0.001), respectively. The administration of caffeine showed a significant decrease to 109% ± 6.2% (*p* < 0.01) and 75% ± 5.9% (*p* < 0.001) for Prx1, and to 99% ± 8.7% (*p* < 0.001) and 104% ± 9.3% (*p* < 0.001) for Srx1 to both times examined. Interestingly, there was a reduction in Prx1 at 48 h under normoxia to 56% ± 2.6% (*p* < 0.001) and an increase of protein expression after 24 h under normoxia to 126% ± 3.6% (*p* < 0.01).

### 2.3. Effects of Caffeine on the Inflammatory Cytokine Expression

**Figure 3:**
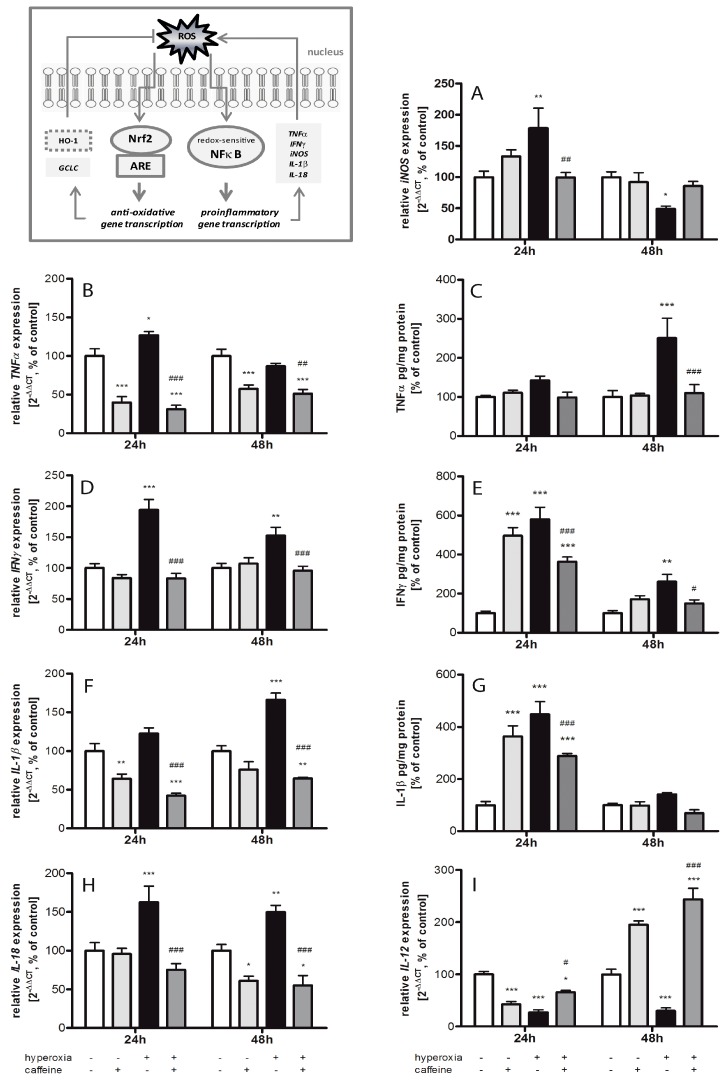
Changes in the expression of cytokines and iNOS in neonatal rat brains after hyperoxic injury with and without caffeine. (**Box**) Nrf2 activation promotes the expression of anti-oxidative gene transcription and reduces oxidative stress-induced inflammatory activation by blocking the redox-sensitive NFκB pathway (ARE; antioxidant response element). The protein concentration (pg/mg protein) and relative mRNA expression of cytokines were measured in brain homogenates from normoxia (**white bars**), caffeine with normoxia (**light grey bars**), hyperoxia (**black bars**), and hyperoxia with caffeine (dark grey bars) by ELISA assay and quantitative real-time PCR of: (**A**) *iNOS*; (**B**,**C**) TNFα; (**D**,**E**) IFNγ; (**F**,**G**) IL-1β; (**H**) *IL-18*; and (**I**) *IL-12*. Data are shown as mean ± SEM, *n* = 4–5 per group per time point. The 100% value is: (**A**) 1.016 and 1.015 *C*_T_; (**B**) 1.021 and 1.018 *C*_T_; (**C**) 21.22 and 29.19 pg/mg protein; (**D**) 1.012 and 1.013 *C*_T_; (**E**) 1.701 and 2.121 pg/mg protein; (**F**) 1.021 and 1.010 *C*_T_; (**G**) 16.58 and 8.37 pg/mg protein; (**H**) 1.026 and 1.016 *C*_T_; and (**I**) 1.006 and 1.019 *C*_T_ for 24 and 48 h groups, respectively. Data were analyzed by two-way ANOVA with Bonferroni post hoc test, with * *p* < 0.05, ** *p* < 0.01, and *** *p* < 0.001 versus control (atmospheric air), and ^#^ *p* < 0.05, ^##^ *p* < 0.01, and ^###^ *p* < 0.001 versus hyperoxia (80% oxygen without caffeine).

The cerebral neuro-inflammatory response in our neonatal oxidative stress model was analyzed by measuring changes in cytokine production and inducible nitric oxide synthase (iNOS) in the brain. Using qPCR for gene expression and ELISA for protein expression analysis, we determined levels of tumor necrosis factor α (TNFα), interleukin (IL)-1β, interferon γ (IFNγ), and IL-18 as pro-inflammatory cytokines, IL-12 as early pro- and late anti-inflammatory cytokine, and iNOS. Overall, significant differences between normoxia control group and the hyperoxia group were detected for all cytokines at 24 and/or 48 h (Figure 3).

Oxidative stress is usually thought to be responsible for tissue injury associated with a range of brain injury, inflammation, and degenerative processes. Moreover, inflammatory target proteins, including iNOS, are associated with oxidative stress induced by pro-inflammatory factors such as cytokines. Expression of *iNOS* (Figure 3A) was significantly increased by 24 h hyperoxia (179% ± 32.1%; *p* < 0.01), and significantly decreased at 48 h (49% ± 4.5%; *p* < 0.05), which was blocked by caffeine at 24 h (100% ± 8.0%; *p* < 0.01).

In response to brain injury by hyperoxia, TNFα production is rapidly increased. An increase of *TNFα* mRNA (127% ± 4.8%; *p* < 0.05) was found after 24 h of hyperoxia (Figure 3B). After 48 h of hyperoxia, the increase in protein expression of TNFα (Figure 3C) was largely pronounced (251% ± 50.8%; *p* < 0.001). The administration of caffeine drastically reduced *TNFα* mRNA expression under hyperoxic conditions at 24 h (31% ± 5.2%; *p* < 0.001) and at 48 h (51% ± 5.5%; *p* < 0.01), which was also observed under normoxic conditions (at 24 h 40% ± 7.6%; *p* < 0.001; at 48 h 57% ± 5.1%; *p* < 0.001). On translational protein levels, caffeine caused a marked reduction in the expression of TNFα protein at 48 h (from 251% ± 50.8% to 110% ± 21.7%; *p* < 0.001). However, TNFα protein was not affected by caffeine in newborn rats kept at atmospheric air (Figure 3C).

In relation to the cytokines measured in these experiments, IFNγ showed the largest changes in mRNA and protein levels in the hyperoxic brain (Figure 3D,E). A significant increase was observed after 24 h (mRNA 194% ± 16.8%, *p* < 0.001; protein 580% ± 61.6%, *p* < 0.001) and 48 h (mRNA 153% ± 13.3%, *p* < 0.01; protein 262% ± 36.7%, *p* < 0.01) of hyperoxia exposure. A single dose of caffeine diminished this expression at both time points. At 24 and 48 h, there is a significant reduction in protein expression to 363% ± 24.1% (*p* < 0.001) and to 150% ± 18.5% (*p* < 0.05), respectively. Similarly, caffeine affects under hyperoxia *IFNγ* on the mRNA level with an attenuation to 83% ± 8.3% (*p* < 0.001) after 24 h and 96% ± 6.8% (*p* < 0.001) after 48 h. Remarkably, administration of caffeine in control animals showed a high increase of the IFNγ protein expression at 24 h (497% ± 40.8%; *p* < 0.001) compared to untreated control litters, whereas no changes were observed at the mRNA level.

As a further indication of pro-inflammatory responses, 24 h hyperoxia caused a large increase of IL-1β protein expression (448% ± 48.6%; *p* < 0.001) as compared to controls (Figure 3F,G). The application of caffeine in hyperoxic animal led to a reduction of IL-1β protein (at 24 h 289% ± 9.4%; *p* < 0.001). Caffeine administration alone under normoxia also showed a dramatic increase in the protein level after 24 h (363% ± 40.5%; *p* < 0.001). The mRNA expression significantly decreased during normoxia and caffeine administration after 24 h (64% ± 5.9%; *p* < 0.01). Hyperoxia exposure resulted in a non-significant increase (122% ± 7.4%) at 24 h, and a significant increase after 48 h hyperoxia exposure (166% ± 8.7%; *p* < 0.001). Here also, mRNA expression under hyperoxia is significantly reduced below the normoxic level by caffeine administration at 24 h (42% ± 3.2%; *p* < 0.001) and 48 h (65% ± 1.7%; *p* < 0.001).

Exposure to hyperoxia increased the concentration of *IL-18* mRNA at 24 h to 163% ± 20.6% (*p* < 0.001) and 48 h to 150% ± 8.6% (*p* < 0.01) compared with rat pups exposed to normoxia (Figure 3H). The application of caffeine significantly reduced cytokine level below hyperoxia at both time points to 72% ± 7.9% (*p* < 0.001) and to 55% ± 12.6% (*p* < 0.001), respectively. Caffeine under non-hyperoxic conditions resulted in a significant reduction in *IL-18* expression at 48 h (61% ± 5.9%; *p* < 0.05).

Interestingly, a drastic reduction in *IL-12* mRNA expression (Figure 3I; 28% ± 4.7%; *p* < 0.001, and 30% ± 5.7%; *p* < 0.001) is detected after 24 and 48 h of hyperoxia, respectively. Under hyperoxia exposure with prior application of caffeine, the expression increased significantly at 24 h at 66% ± 3.6% (*p* < 0.05) and at 48 h at 244% ± 21.5% (*p* < 0.001). In contrast, the expression of *IL-12* under normoxia with caffeine was reduced after 24 h (43% ± 5.3%; *p* < 0.001) and increased after 48 h (195% ± 7.5%; *p* < 0.001).

### 2.4. Hyperoxia Modulates Gene Expression of Transcription Factors and Caffeine Counteracts

**Figure 4:**
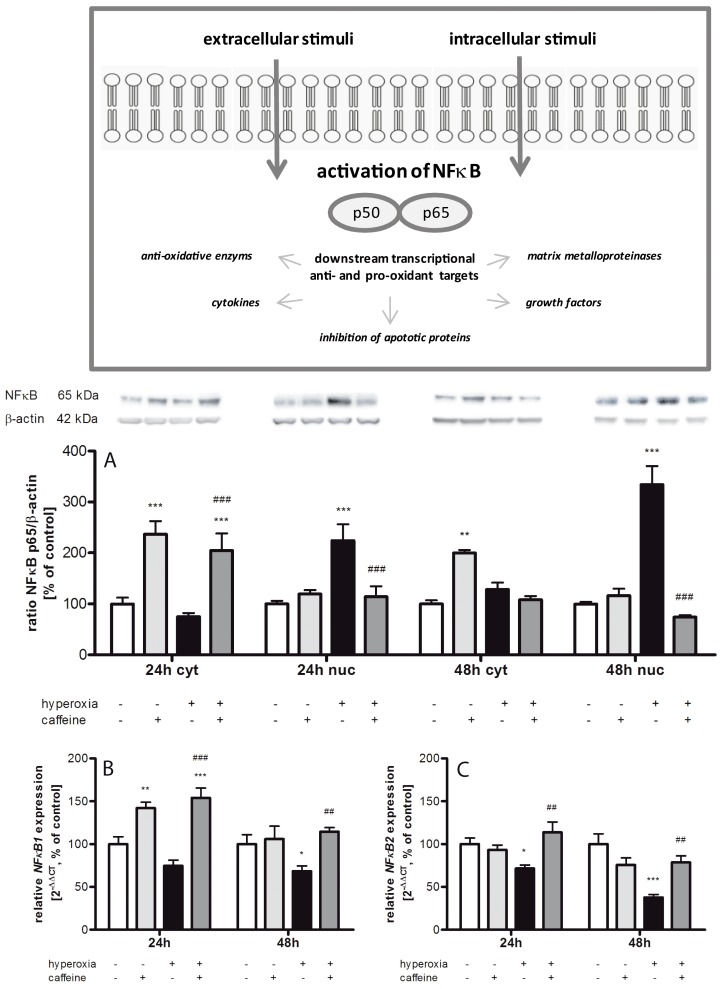
NFκB expression is affected by caffeine administration. (**Box**) Stimulation of the NFκB pathway is mediated by diverse signal transduction cascades. The change of protein expression of NFκB were measured in brain homogenates of normoxia (**white bars**), caffeine with normoxia (**light grey bars**), hyperoxia (**black bars**), and hyperoxia with caffeine (dark grey bars) by Western blot and quantitative real-time PCR with: (**A**) NFκB protein expression in cytosolic (cyt) and nuclear (nuc) fraction; and (**B**,**C**) *NF*κ*B* mRNA expression after 24 h and 48 h of oxygen exposure. Data are shown as mean ± SEM, *n* = 5 per group per time point. The 100% value is: (**A**) (cyt) 0.035 and 0.024 ratio intensity/mm^2^ and (nuc) 0.092 and 0.127 ratio intensity/mm^2^; (**B**) 1.017 and 1.028 *C*_T_; and (**C**) 1.010 and 1.032 *C*_T_ for 24 and 48 h groups, respectively. Data were analyzed by two-way ANOVA with Bonferroni post hoc test, with * *p* < 0.05, ** *p* < 0.01, and *** *p* < 0.001 versus control (atmospheric air), and ^##^ *p* < 0.01 and ^###^ *p* < 0.001 versus hyperoxia (80% oxygen without caffeine).

As shown in Figure 4A the nuclear factor of kappa light polypeptide gene enhancer in B-cells (NFκB) protein expression was increased by caffeine under normoxia/hyperoxia at 24 h (237% ± 25.8%; *p* < 0.001, and 204% ± 36.7%; *p* < 0.001, respectively) and at 48 h (200% ± 6.0%; *p* < 0.01) in the cytosol compared to the control group. There were no changes under hyperoxic exposure alone, while the alteration in protein level in the nucleus indicated an up-regulation of NFκB under hyperoxic condition at both time points (224% ± 31.9%; *p* < 0.001, and 334% ± 36.3%; *p* < 0.001, respectively). In the nuclear protein fraction, a single dose of caffeine significantly reduced NFκB expression at 24 h to 114% ± 20.8% (*p* < 0.001), and at 48 h to 74% ± 4.1% (*p* < 0.001).

The mRNA expression ratios of *NF*κ*B1* and *NF*κ*B2* corresponded with the cytosolic protein data (Figure 4B,C). Here we showed that hyperoxia resulted in an mRNA reduction of both *NFκB* forms (*NFκB1* and *2*) at both time points. Caffeine with hyperoxia exposure always led to an increase in mRNA expression (*NFκB1* at 24 h from 75% ± 6.5% to 154% ± 11.3% (*p* < 0.001), and at 48 h from 68% ± 6.1% to 115% ± 4.7% (*p* < 0.01); *NFκB2* at 24 h from 72% ± 4.0% to 114% ± 12.1% (*p* < 0.01), and at 48 h from 38% ± 3.5% to 79% ± 7.7% (*p* < 0.01)). *NFκB1* expression was bolstered by caffeine under normoxia after 24 h (142% ± 6.8%; *p* < 0.01).

### 2.5. Caffeine Prevents Hyperoxia-Mediated Increase in Apoptotic Gene Expression

**Figure 5:**
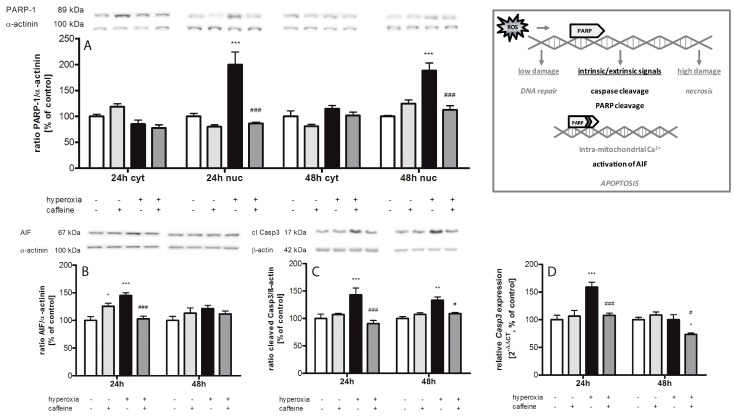
Caffeine inhibits hyperoxia-induced apoptotic key mediators. (**Box**) ROS-induced DNA damage activated PARP-1, an initially repair enzyme. PARP-1 is a substrate of caspase-3, a member of a highly specialized family of cysteinyl-aspartate proteases (caspases) involved in apoptosis. Upon cleavage and progression of cell death intramitochondrial calcium is released and AIF is induced. Analysis of the expression of apoptotic mediators were conducted in cytosolic (cyt) and nuclear (nuc) protein fraction and relative mRNA expression was measured in brain homogenates of normoxia (white bars), caffeine with normoxia (light grey bars), hyperoxia (black bars), and hyperoxia with caffeine (dark grey bars) by Western blot and quantitative real-time PCR with protein expression of: (**A**) cleaved PARP-1 (cyt and nuc); (**B**) AIF (cyt); and (**C**) cleaved caspase-3 (cyt); and mRNA expression of (**D**) *caspase-3*. Data are shown as mean ± SEM, *n* = 5 per group per time point. The 100% value is: (**A**) (cyt) 0.051 and 0.760 ratio intensity/mm^2^ and (nuc) 0.064 and 0.043 ratio intensity/mm^2^; (**B**) 0.216 and 0.309 ratio intensity/mm^2^; (**C**) 0.691 and 0.823 ratio intensity/mm^2^; and (**D**) 1.014 and 1.004 C_T_ for 24 h and 48 h groups, respectively. Data were analyzed by two-way ANOVA with Bonferroni post hoc test, with * *p* < 0.05, ** *p* < 0.01, and *** *p* < 0.001 versus control (atmospheric air), and ^#^ *p* < 0.05, and ^###^ *p* < 0.001 versus hyperoxia (80% oxygen without caffeine).

We measured the cytosolic and nuclear expression of cleaved poly (ADP-ribose) polymerase-1 (PARP-1) (Figure 5A). Hyperoxia increased nuclear PARP-1 at both times measured (24 h with 200% ± 24.3%; *p* < 0.001; 48 h with 188% ± 14.5%; *p* < 0.001) and no changes observed in cytosolic fraction. Caffeine reduced this increase to the normoxic level (*p* < 0.001). High oxygen also induced cytosolic apoptosis inducing factor (AIF) protein at 24 h (Figure 5B; 145% ± 4.9%; *p* < 0.001), increased caspase-3 cleavage (Figure 5C) at 24 h (143% ± 12.3%; *p* < 0.001) and at 48 h (133% ± 6.0%; *p* < 0.01), and enhanced *caspase-3* mRNA expression at 24 h (Figure 5D; 159% ± 9.0%; *p* < 0.001). For the targets investigated, the application of caffeine resulted in a significant reduction of protein and/or mRNA expression (AIF at 24 h from 145% ± 4.9% to 103% ± 4.6% (*p* < 0.001); cleaved caspase-3 at 24 h from 143% ± 12.3% to 91% ± 5.6% (*p* < 0.001), and at 48 h from 133% ± 6.0% to 109% ± 2.0% (*p* < 0.05); *caspase-3* mRNA expression at 24 h from 159% ± 9.0% to 108% ± 3.7% (*p* < 0.001), and at 48 h from to 74% ± 2.6% (*p* < 0.05)).

### 2.6. Caffeine Effects on Matrix Metalloproteinases and the tPa/Plasminogen System

**Figure 6:**
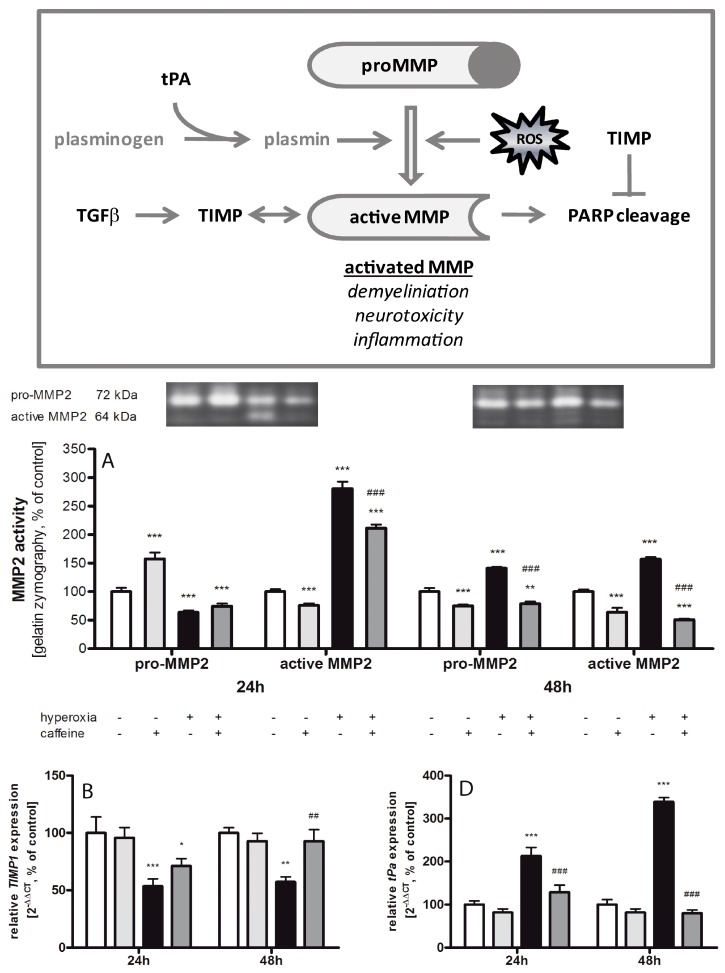

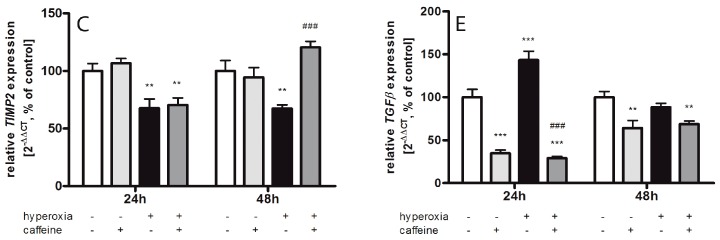
Hyperoxia-mediated imbalance in the MMP-TIMP system counteracted by caffeine. (**Box**) The matrix metalloproteinase (MMP) system can be activated via the plasminogen/plasmin system, and ROS. Active MMPs affect a variety of extracellular and immune regulatory proteins and are involved in modulating and degrading processes. Active MMPs can be inhibited by tissue inhibitors of metalloproteinases (TIMPs). TGF-β can enhance the expression of MMPs and TIMPs. MMP2 and MMP9 are able to cleave PARP-1. The MMP inhibitor TIMP2 is able to block PARP-1 degradation. The pro and active MMPs were analyzed with gelatin zymography and relative mRNA expression were measured in brain homogenates of normoxia (**white bars**), caffeine with normoxia (light grey bars), hyperoxia (**black bars**), and hyperoxia with caffeine (**dark grey bars**) by quantitative real-time PCR of: (**A**) pro/active MMP2; (**B**,**C**) *TIMP1/2* mRNA; (**D**) *tissue plasminogen activator* (*tPa*) mRNA; and (**E**) *TGF-β* mRNA. Data are shown as mean ± SEM, *n* = 4 (for gelatin zymography)-5 per group per time point. The 100% value is (**A**) (pro) 7.849 × 10^6^ and 0.949 × 10^6^ intensity/mm^2^ and (active) 11.18 × 10^6^ and 0.731 × 10^6^ intensity/mm^2^; (**B**) 1.043 and 1.004 *C*_T_; (**C**) 1.008 and 1.017 *C*_T_; (**D**) 1.013 and 1.026 *C*_T_; and (**E**) 1.018 and 1.010 *C*_T_ for 24 and 48 h groups, respectively. Data were analyzed by two-way ANOVA with Bonferroni post hoc test, with * *p* < 0.05, ** *p* < 0.01, and *** *p* < 0.001 versus control (atmospheric air), and ^##^ *p* < 0.01 and ^###^ *p* < 0.001 versus hyperoxia (80% oxygen without caffeine).

Gel gelatin zymography was used to investigate the effect of oxidative stress and caffeine on the gelatinases expressions (Figure 6A). In the hyperoxic group a reduced level of pro-matrix metalloproteinases (MMP) 2 (72 kDa) was detected at 24 h (64% ± 3.1%; *p* < 0.001). After 48 h hyperoxia pro-MMP2 was increased (141% ± 2.2%; *p* < 0.001) and significantly decreased with caffeine application (79% ± 1.9%; *p* < 0.001). Caffeine alone under normoxic exposure showed a higher level on pro-MMP2 after 24 h (157% ± 5.6%; *p* < 0.001) and a lower level after 48 h (75% ± 1.2%; *p* < 0.001). At both time points acute hyperoxia exposure led to a drastic increase of active MMP2 (24 h with 281% ± 11.9%; *p* < 0.001; 48 h with 157% ± 3.7%; *p* < 0.001), which was again mitigated by caffeine (24 h to 211% ± 3.2%; *p* < 0.001; 48 h to 51% ± 1.0%; *p* < 0.001). Caffeine application at normoxia reduced the processing of active MMP2 (24 h to 76% ± 1.8%; *p* < 0.001; 48 h to 75% ± 1.2%; *p* < 0.001). Contrary to MMP2, no active MMP9 band was detected in any samples.

The inhibitors of these metalloproteinases, especially tissue inhibitor of metalloproteinase (TIMP) 1/2 (Figure 6B,C), are down-regulated at 24 h (*TIMP1*: 54% ± 6.5%; *p* < 0.001; *TIMP2*: 68% ± 7.9%; *p* < 0.01) and 48 h (*TIMP1*: 57% ± 4.6%; *p* < 0.01; *TIMP2*: 67% ± 3.2%; *p* < 0.01). Caffeine increased the mRNA expression of both inhibitors only at 48 h (*TIMP1*: 93% ± 10.2%; *p* < 0.01; *TIMP2*: 121% ± 5.2%; *p* < 0.001). Caffeine alone had no significant influence. The tissue plasminogen activator (tPa) generates the active protease, plasmin, which is capable of degrading numerous substrates. Here (Figure 6D), hyperoxia led to a strong increase of *tPa* mRNA expression (24 h to 213% ± 19.9%; *p* < 0.001; 48 h to 339% ± 9.6%; *p* < 0.001) and a single dose of caffeine reduced *tPa* to normoxia level (24 h to 129% ± 16.6%; *p* < 0.001; 48 h to 80% ± 7.5%; *p* < 0.001).

The gene expression of *transforming growth factor*-(*TGF)-β* (Figure 6E) is highly increased after 24 h (143% ± 10.4%; *p* < 0.001) of hyperoxia. Caffeine strongly reduced *TGF-β* mRNA under normoxia (24 h to 35% ± 3.8%; *p* < 0.001; 48 h to 64% ± 8.8%; *p* < 0.01) as compared to control, as well as hyperoxic conditions compared to the hyperoxic group without caffeine (24 h to 29% ± 1.9%; *p* < 0.001; 48 h to 69% ± 3.6%; *p* < 0.01) at both time points.

## 3. Discussion

Neuronal injury and neurological maldevelopment in preterm infants is commonly ascribed to perinatal infection/inflammation and to oxidative stress hitting the immature brain at a vulnerable phase of development. In our neonatal rat model, oxidative stress is induced by early exposure to high oxygen levels for 24 and 48 h, in which neural cell injury has been shown to occur in a way that is comparable to the human neonatal situation [32].

In preterm infants, caffeine is used to stimulate breathing activity and prevent the onset of apnoea [24]. Based on some clinical studies, benefits of caffeine administration for the neurological outcome of preterm infants have been suggested [13,14,21,24]. To date, the mechanisms of neuroprotection afforded by caffeine are under investigated. Our study of the neonatal oxidative stress model elucidate that a single dose of caffeine in an acute hyperoxia model effectively inhibits pro-inflammatory cytokine production and pro-apoptotic effectors, modulates anti-oxidative enzymes, and affects components of the extracellular matrix.

### 3.1. Oxidative Stress Response

Oxidative stress is caused by excess of free radical formation and/or an overproduction of ROS, and is a major factor of injury in the developing brain leading to neurological sequelae of preterm birth [33,34]. In our study in six-day-old hyperoxia-exposed rats, oxidative stress was found to be caused by an increase in hydrogen peroxide, a reactive oxygen metabolic product and a major regulator of oxidative stress-related processes, and of the stress response protein HO-1, which confers protection against a variety of oxidant-induced cell and tissue injury [35], leading to an increase in lipid peroxidation [36].

The increased HO-1 expression suggests an activation of protective mechanisms in response to unwanted cellular oxidative conditions [35]. Anti-oxidative enzymes, such as glutathione peroxidase, catalase, and SODs are essential to preserving cells from exposure to oxidative damage [37]. SOD converts superoxide into hydrogen peroxide and oxygen, with hydrogen peroxide being less toxic. Several studies have demonstrated increases in oxidative stress markers after exposure to hyperoxia in animals and humans [4,38]. Changes in the gene expression and enzymatic activity of SODs have been characterized in the rodent brain as a consequence of aging, oxygen, or pro-oxidant drug exposure [39]. Our results revealed a drastic oxygen-induced reduction in SOD3 transcript in newborn rat brains. These findings are contrary to previous studies which showed an oxidative stress-induced up-regulation of the SOD transcript, but SOD activity was unaffected [37].

Nrf2 is a redox-sensitive transcription factor that mediates protection against oxidative stress via the transcriptional activation of several antioxidant enzymes through the antioxidant response element (ARE). Nrf2 is negatively regulated by Keap1 thereby providing inducible antioxidant defense. Under basal conditions Nrf2 is regulated by Keap1, but under oxidative stress, the Nrf2 pathways can also be regulated independently of Keap1 [40]. The experimentally determined *Nrf2* mRNA increase confirms the induction of antioxidant genes under hyperoxic conditions while the expression of Keap1 is unchanged.

Glutathione (GSH) plays an important role for antioxidant defense and for the regulation of intracellular redox homeostasis. The rate-limiting step of cellular antioxidant GSH is catalyzed by glutamate-cysteine ligase (GCL), which consists of a catalytic (GCLC) and a modifier (GCLM) subunit [41]. GCLC is regulated by Nrf2 via NFκB pathway [42]. Hyperoxia demonstrated a significant induction of GCLC mRNA expression in the developing brain. Together with the hyperoxia-induced increase of Nrf2 expression, these results underline the interplay of antioxidant responses [43].

Peroxiredoxin (Prx) and sulfiredoxin (Srx) are important proteins of the thioredoxin family and a key regulator of genes involved in oxidant defense, such as SOD3, Prx1, Srx1, and HO-1, and redox signaling. It is known that non-physiological oxygen concentrations change the balance of the Srx/Prx system in the developing brain [44]. Prx and Srx can act as an antioxidant and Srx solely reduces over-oxidized typical 2-Cys-Prx [45]. Hyperoxic changes elicit reactions of the antioxidant enzyme system, specifically the SODs, which play an essential role in antioxidant defense [37]. Our current data confirm previous studies [44] showing under oxidative stress a significant increase of peroxide reducing protein Prx1 and of antioxidant Srx1 protein expression.

An antioxidant activity of caffeine has been assumed in recent studies in which neuroprotection was demonstrated in patients suffering from neurodegenerative disorders [11,12]. In our study using acute hyperoxia, we demonstrated a highly significant anti-oxidative property of caffeine. A single dose of caffeine resulted in first line in a reduction of the oxidative response of lipid peroxidation, a marker of oxidative stress [46], in generation of hydrogen peroxide (H_2_O_2_), and in the relevant context the expression of HO-1 and *Nrf2*. In the second line, caffeine improved *SOD* RNA expression and suppressed Prx1 and Srx1 protein expressions. These results point to a broad interaction of caffeine with the antioxidant defense network. The reduced *SOD* expression under hyperoxic conditions could be explained by an exhaustion of the anti-oxidant system which was successfully counteracted by caffeine. Ahotupa et al. showed an initial decrease of SOD in hyperoxic rat brains, followed by a slight induction of its activity at a later time point [47]. Altogether, the findings suggest that the antioxidant defense capacity is diminished during exposure to hyperoxia and, moreover, represents a promising target for protective pharmacological strategies.

### 3.2. Inflammatory Response via NFκB Pathway

Dependent on the level of generated ROS, different redox-sensitive transcription factors are activated. Moderate oxidative stress is related to Nrf2 activation and intermediate amount of ROS initiates an inflammatory response through activation of NFκB pathway [48]. Our results in the developing brain underline a connection between induced Nrf2 expression and increased protein expression of HO-1, Prx1, and Srx1. Moreover, caffeine treatment seems to modify the redox-sensitive response on the Nrf2/Keap1 pathway, since it attenuated the hyperoxia-induced increase of NFκB expression. NFκB is activated by different endogenous stimuli, as well as by oxidative stress or cytokines [49,50]. In our hyperoxic rat pups, the pro-inflammatory genes TNFα, IFNγ, *iNOS*, IL-1β, and *IL-18* were induced. An interaction of oxidative stress and inflammatory responses has previously been described [51]. The pro-inflammatory cytokine IL-18 plays an essential role in IFNγ induction [52] and is associated with cell death after hyperoxia insult [38]. Excessive NO production causes tissues damage by formation of peroxynitrite. Consequently, nitrotyrosine is being formed and lipid peroxidation gets initiated [53]. Oxidative stress may enhance expression of the inducible isoform of NOS (iNOS) through the activation of NFκB [54]. Activation of NFκB may also increase the release of pro-inflammatory cytokines which again can induce iNOS, thus resulting in enhanced production of NO. Interestingly, Hoehn et al. [55] demonstrated that hyperoxia induces up-regulation of iNOS in the immature rat brain as a possible cause of cellular damage in the immature brain [56].

In our study, caffeine significantly reduced inflammatory responses. Surprisingly, *IL-12* mRNA expression was dramatically reduced under hyperoxia, which was blocked by caffeine. IL-12 is produced by microglia [57], and pro- and anti-inflammatory effects are reported [58,59]. It has to be discussed that caffeine not only exerted beneficial actions in hyperoxic rats but also triggered cellular changes and pro-inflammatory responses in normoxia control rats [60]. Caffeine reduced lipid peroxidation not only in response to hyperoxia but also under normoxic conditions. At the same time, the transcripts of *SOD2*/*3* and protein expression of Srx1 and pro-inflammatory cytokines (IL-1β and IFNγ) increased after caffeine administration at normoxia. Leon-Carmona and Galano disclosed in their functional studies that caffeine may act as radical scavenger [61]. Caffeine also reduced hydrogen levels in lung cells in vitro [62]. In mice, caffeine reduces lipid peroxidation [63]. Cytokine and anti-oxidative enzyme over expression in normoxic exposure could be protective in oxidative stress models [64,65]. Chavez-Valdez and co-workers discussed a range of caffeine plasma level appropriate for patient therapy, and higher plasma levels outside of these range coincided with an increase of inflammatory cytokines in a clinical study with preterm infants [18].

### 3.3. Apoptosis

High levels of ROS have been shown to induce apoptosis in the immature brain after hyperoxia [32]. In our study, exposure to high oxygen induced the nuclear acting of PARP-1, an enzyme which is activated by DNA strand breaks, and the caspase-independent AIF, and increased cleavage of caspase-3. In previous studies, exposure to postnatal hyperoxia also caused an increase in apoptotic cell death [16,44,51,66]. Anti-apoptotic properties of caffeine, also previously demonstrated by our group [16], were seen in this study by modified levels of PARP-1, AIF, and cleaved caspase-3 levels. In a hypoxic ischemic model of young rats, caffeine also revealed apoptosis-inhibiting effects [67]. Attenuation of PARP-1 inhibiting capacity has also been attributed to caffeine metabolites [68]. In our study, elevated PARP-1 and AIF were also found in controls after injection of 10 mg/kg caffeine. An increase of caspase-3 positive cells was also reported in sham animals of a hypoxia-ischemia study that received 20 mg/kg caffeine [67]. It seems possible that regulation of neuronal cell survival by caffeine occurs via direct activation of the NFκB pathway [69].

### 3.4. Fibrinolytic and Matrix Metalloproteinases System

Free radicals and ROS seem to activate matrix metalloproteinases (MMPs), with the biological function to degrade extracellular matrix of cerebral blood vessels and neurons [70], and active MMPs may disrupt the vital blood-brain barrier. Hyperoxia led to a drastic increase of MMP2 activation, while tissue inhibitors of MMPs (TIMP)-2, decreased. Caffeine might inhibit the processing of MMP2 through inhibition of TIMP2 expression, but molecular protection might also start further upstream through reduction of *tPa* transcript expression as found in our study, thus leading to less processing of active MMP. TGF-β is a multifunctional cytokine that regulates a broad diversity of physiological and pathological processes. Plasmin releases latent forms of growth factors such as TGF-β [71]. TGF-β effects on matrix degeneration are characterized by mixed reciprocally action of MMPs and inhibition of TIMPs to reduce the MMP/TIMP balance [72], so caffeine minimizes TGF-β expression. Sifringer et al. showed in traumatic and hyperoxic brain injury models a time-dependent increase of MMP activation in correlation to *TIMP* mRNA expression, and active MMP2 and MMP9 were reduced by protective treatment with erythropoietin [73].

### 3.5. Caffeine and Neuroprotection

For the clinical situation, it is important to precisely define the effects of caffeine on the otherwise healthy brain in order to avoid harm in preterm infants. The increase of apoptotic factors in normoxic newborn pups coincides with higher activity of MMP2 and also with increased NFκB protein expression in these animals. Since the injection of caffeine was performed 15 min before the beginning of hyperoxia exposure, it is possible that a certain degree of cellular stimulation is caused by caffeine that functions as a protective preconditioning in those animals challenged by hyperoxia later on. Preconditioning has been characterized as a protective procedure in brain injury models involving inflammatory stimulation [74], ischemia [75], and hypoxia [76] prior to the injurious events. Ischemic preconditioning is currently under evaluation for open heart surgery in various clinical centers and studies [77,78]. It is possible that caffeine represents a drug that in addition to its anti-oxidant and anti-inflammatory properties might also be useful for protective preconditioning of the brain. For prevention of brain injury in preterm infants, it has been suggested to investigate the use of anti-oxidant compounds such as melatonin, acetylcysteine, or allopurinol [79]. The strategy to use caffeine for brain protection in preterm infants would provide great advantages of a drug being licensed for this vulnerable patient population. The pharmacological properties and safety aspects of caffeine are well known to clinicians taking care of preterm infants, and ethical issues for clinical trials or routine care administration will be minor as compared to most other drug candidates.

## 4. Materials and Methods

### 4.1. Animals and Study Design

All procedures were approved by the state animal welfare authorities (LAGeSo G-0307/09) and followed institutional guidelines. Six-day-old *Wistar rats* from time-pregnant dams were obtained from Charité-Universitätsmedizin Berlin (Germany) and randomly assigned to cages and treatment. Animals were housed under controlled temperature and light conditions with food and water ad libitum. Experimental procedure and caffeine administration were carried out as described previously [16]. The rat pups were divided into two overarching experimental groups. From Postnatal Day 6 (P6), rat pups and their dams in all experimental groups were exposed to either 80% oxygen (hyperoxia, OxyCycler BioSpherix, Lacona, NY, USA) or atmospheric air (normoxia). In relation to the exposure to oxygen, the pups were divided into further subgroups, depending on the drug administration. Neonatal rats were administered vehicle (0.9% saline) or caffeine (10 mg/kg) as single application. Caffeine (Sigma-Aldrich, Steinheim, Germany) was dissolved in sterile distillated water. Each experimental group consisted of five animals with mixed gender. All injections of drug and vehicle were given intraperitoneally (i.p.) as a fixed proportion of body weight (100 µL/10 g). Caffeine or saline were administrated once 15 min before the start of atmospheric air or oxygen exposure.

### 4.2. Tissue Preparation

After 24 h (P7) or 48 h (P8) of exposure, rats were transcardially perfused with normal saline (pH 7.4) under anesthesia (i.p.) of ketamine (50 mg/kg), xylazine (10 mg/kg), and acepromazine (2 mg/kg), then decapitated, the olfactory bulb and cerebellum were removed, and brain hemispheres were snap-frozen in liquid nitrogen and stored at −80 °C.

### 4.3. Protein Extraction

Protein was extracted as described in [16,44]. Briefly, snap-frozen brain tissue was homogenized in RIPA buffer solution for protein extraction. The homogenate was centrifuged at 3000× *g* (4 °C) for 10 min, the microsomal fraction was subsequently centrifuged at 17,000× *g* (4 °C) for 20 min, and stored at −80 °C until further analysis. After collecting the supernatant, protein concentrations were determined using the Pierce BCA kit (Pierce/Thermo Scientific, Rockford, IL, USA) with 30 min incubation at 37 °C prior to spectrophotometry at 562 nm.

### 4.4. Immunoblotting

Western blotting was performed as previously described [16,66] for following proteins: apoptosis inducing factor (AIF), cleaved caspase-3 (cleaved Casp3), nuclear factor of kappa light polypeptide gene enhancer in B-cells (NFκB), poly (ADP-ribose) polymerase-1 (PARP-1), peroxiredoxin 1 (Prx1), and sulfiredoxin 1 (Srx1). Briefly, protein extracts (20 µg per sample) were denatured in Laemmli sample loading buffer at 95 °C, size-fractionated by 12.5% (for AIF, NFκB, PARP-1, and Prx1) or 15% (for cleaved casp3 and Srx1) sodium dodecyl sulfate (SDS) polyacrylamide gel electrophoresis and electro transferred in transfer buffer to a nitrocellulose membrane (0.2 µm pore, Bio-Rad, Munich, Germany), or for NFκB to a PVDF membrane (0.45 µm pore, Merck Millipore, Darmstadt, Germany). Membranes were blocked for 1 h at room temperature (Srx1 and Prx1 with 1% (*v*/*v*) horse serum in Tris-buffered saline /0.1% (*v*/*v*) Tween 20 (TBST); AIF, cleaved Casp3, NFκB, and PARP-1 with 5% (*w*/*v*) bovine serum albumin (Serva, Heidelberg, Germany) in TBST). Equal loading and transfer of proteins was confirmed by staining the membranes with Ponceau S solution (Fluka, Buchs, Switzerland). The membranes were incubated overnight at 4 °C with the following antibodies: rabbit polyclonal anti-AIF (67 kDa; 1:1000; Cell Signaling, Cambridge, UK), rabbit monoclonal anti-cleaved caspase-3 (17 kDa; 1:1000; Cell Signaling), rabbit polyclonal anti-NFκB p65 (~60 kDa, 1:2500 for cytosolic and 1:750 for nuclear protein fraction; Merck Millipore), rabbit monoclonal anti-PARP-1 (89 kDa; 1:1000; Cell Signaling), goat polyclonal anti-peroxiredoxin 1 (Prx1; 50 kDa dimer; 1:400; Santa Cruz Biotechnology, Heidelberg, Germany), polyclonal goat anti-sulfiredoxin 1 (Srx1; 13 kDa; 1:200; Santa Cruz Biotechnology) diluted in 0.5% (*v*/*v*) horse serum or 1% (*w*/*v*) bovine serum albumin in TBST, corresponding to the blocking solution. As reference controls, were used monoclonal mouse anti-β-actin (42 kDa; 1:10,000; Sigma-Aldrich) and rabbit polyclonal anti-α-actinin (100 kDa; 1:1000; Cell Signaling) diluted in 1% (*w*/*v*) bovine serum albumin in TBST. Secondary incubations were performed with horseradish peroxidase-linked polyclonal donkey anti-goat (1:3000; Dianova, Hamburg, Germany), polyclonal rabbit anti-mouse (1:1000; DAKO, Glostrup, Denmark), or polyclonal donkey anti-rabbit (1:4000; Dianova) antibodies, diluted in 1% (*w*/*v*) bovine serum albumin in TBST, respectively. Positive signals were visualized using enhanced chemiluminescence (ECL; Amersham Biosciences, Freiburg, Germany) and quantified using a ChemiDoc™ XRS+ system and the software Image Lab™ (Bio-Rad). Each experiment was repeated three times.

### 4.5. Gelatin Zymography

Snap-frozen tissue was homogenized in working buffer (150 mM NaCl, 5 mM CaCl_2_, 0.05% (*v*/*v*) Brij 35, 1% (*v*/*v*) Triton X-100, 0.02% (*w*/*v*) NaN_3_, 50 mM Tris-HCl pH 7.4) on ice, followed by centrifugation for 5 min at 12,000× *g*. The protein concentration of the supernatant was measured with the Pierce BCA kit (Pierce/Thermo Scientific). One milligram of protein was used for enzyme enrichment and purification with Gelatin Sepharose™ 4B (GE Healthcare, Uppsala, Sweden) and eluted in 150 µL working buffer containing 10% (*v*/*v*) dimethylsulfoxide. Protein samples mixed with an equal volume of Novex^®^ SDS-sample buffer (Life Technologies GmbH, Darmstadt, Germany), and incubated for 5 min at room temperature prior to loading to 10% Novex^®^ gelatin-ready zymogram gel (Life Technologies GmbH). Gels were run at 125 V for 2 h. The gels were incubated in renaturation buffer (Novex^®^, Life Technologies GmbH) under gentle agitation for 30 min. They were then equilibrated for 30 min in developing buffer (Novex^®^, Life Technologies GmbH) and incubated in fresh developing buffer at 37 °C overnight, but at least for 18 h. Gels were washed three times with deionized water under gentle agitation for 5 min and stained with Coomassie Blue G 250 (SimplyBlue SafeStain, Life Technologies GmbH) for 30 min, and washed with deionized water for 30 min. Areas of protease activity appear as clear bands against a dark blue background where the protease has digested the gelatin substrate. Quantification of inverted matrix metalloproteinases (MMP) 2 band density was carried out using a ChemiDoc™ XRS+ system and the software Quantity One^®^ (Bio-Rad).

### 4.6. Thiobarbituric Acid Reactive Substances (TBARS) Assay

Concentrations of markers of lipid peroxidation thiobarbituric acid reactive substances (TBARS) was determined using the TBARS assay kit (Cayman Chemical, Ann Arbor, MI, USA) according to manufacturer’s instructions. Briefly, first a mixture of sample or standard and SDS solution was prepared. A color reagent was added to the mixture and boiled for one hour. The reaction was stopped on ice for 10 min and centrifuged at 1600× *g* (4 °C) for 10 min. The supernatant was transferred on a 96 well plate and absorbance was read at 530 nm in a microplate reader. TBARS concentration as a measure for lipid peroxidation was calculated from a malondialdehyde (MDA) standard curve and normalized to the amount of total protein.

### 4.7. Heme Oxygenase-1 (HO-1) Assay

HO-1 concentration was analyzed in samples of brain homogenate using Rat Heme Oxygenase-1 EIA Kit (Precoated, Takara Bio Europe/SAS, Saint-Germain-en-Laye, France) according to manufacturer’s instructions. Briefly, 100 µL of sample or standard was first loaded and incubated for one hour at room temperature. Then the sample solution was removed and the wells washed three times with 400 µL of PBS containing 0.1% (*v*/*v*) Tween 20. Afterwards, 100 µL of antibody-POD conjugate solution was added and incubated at room temperature for one hour. The sample solution was removed and ensuing washed four times with 400 µL of PBS, aspirating thoroughly between washes. Substrate solution (100 µL) was added and incubated for 15 min at room temperature. The reaction was stopped by addition of 100 µL stop solution. The absorbance was read at 450 nm in a microplate reader. HO-1 concentration was determined by comparing sample to the standard curve and values were expressed as nanograms per milligram of protein.

### 4.8. Hydrogen Peroxide Assay

Hydrogen peroxide (H_2_O_2_) was measured in brain homogenates using OxiSelect Hydrogen peroxide assay kit (Cell Biolabs Inc., San Diego, CA, USA) according to manufacturer’s instructions. Briefly, 25 µL of sample or standard was first added to the microtiter plate wells and then mixed thoroughly with 250 µL aqueous working reagent. Afterwards samples were incubated on a shaker for 30 min at room temperature and the absorbance was read at 540 nm. The peroxide content in unknown samples was determined by comparison with a predetermined H_2_O_2_ standard curve.

### 4.9. Enzyme-Linked Immunosorbent Assays (ELISAs)

Tumor necrosis factor α (TNFα), interleukin-1β (IL-1β), and interferon-γ (IFN-γ) concentrations were analyzed in samples of brain homogenate using rat TNFα/TNFSF1A, rat IL-1β/IL-1F2, and rat IFN-γ DuoSet ELISA (R&D Systems GmbH, Wiesbaden-Nordenstadt, Germany) according to manufacturer’s instructions. Briefly, 96-well plates were coated with 100 µL of capture antibody, incubated overnight, washed with 400 µL wash buffer and incubated for 1 h for blocking with 300 µL of reagent diluent. After washing in wash buffer, 100 µL of samples or standards, diluted in reagent diluent, were added and incubation preceded for 2 h. Samples were then washed and incubated for 2 h in the presence of 100 µL of biotinylated detection antibody at room temperature. The supernatants were aspirated, the wells washed and 100 µL of horseradish peroxidase-conjugated streptavidin was added and incubation continued for 20 min. Samples were washed and 100 µL of substrate solution, a 1:1 mixture of H_2_O_2_ and tetramethylbenzidine (R&D Systems GmbH), was added. Samples were incubated in the dark for 20 min and the reaction was stopped using 50 µL 2 N H_2_SO_4_. Plates were read at 450 nm and TNFα, IL-1β, and IFN-γ concentrations were estimated from the standard curve and expressed as picograms per milligram protein.

### 4.10. RNA Extraction and Real-Time PCR

Gene expression analysis was performed as previously described [16,66]. Total RNA was isolated from snap-frozen tissue by acidic phenol/chloroform extraction (peqGOLD RNAPure™; PEQLAB Biotechnologie, Erlangen, Germany) and 2 µg of RNA was reverse transcribed. The PCR products of caspase-3 (*Casp3*), glutamate-cysteine ligase catalytic subunit (*GCLC*), interferon-γ (*IFN-γ*), interleukin-1β (*IL-1β*), interleukin-12b (*IL-12b*), interleukin-18 (*IL-18*), inducible nitric oxide synthase (*iNOS*), Kelch-like ECH-associated protein 1 (*Keap1*), nuclear factor of kappa light polypeptide gene enhancer in B-cells 1 and 2 (*NFκB1* and *NFκB2*), NFE2-related factor 2 (*Nrf2*), superoxide dismutase 1, 2 and 3 (*SOD1*, *SOD2*, and *SOD3*), tissue inhibitor of metalloproteinase 1 and 2 (*TIMP1* and *TIMP2*), tumor necrosis factor α (*TNFα*), tissue plasminogen activator (*tPa*), and hypoxanthine-guanine phosphoribosyltransferase (*HPRT*) were quantified in real time, using dye-labeled fluorogenic reporter oligonucleotide probes and primers (Table 1).

The FAM spectral data were collected from reactions carried out in separate tubes using the same stock of cDNA to avoid spectral overlap between FAM/TAMRA and limitations of reagents. PCR and detection were performed in triplicate and repeated two times for each sample in 11 µL reaction mix, which contained 5 µL of 2× KAPA PROBE FAST qPCR Master mix (PEQLAB Biotechnologie), 2.5 µL of 1.25 µM oligonucleotide mix, 0.5 µL (0.5 µM) of probe (BioTeZ, Berlin, Germany), and 3 to 17 ng of cDNA template with *HPRT* used as an internal reference. The PCR amplification was performed in 96-well optical reaction plates for 40 cycles with each cycle at 94 °C for 15 s and 60 °C for 1 min. The expression of target genes was analyzed with the StepOnePlus real-time PCR system (Life Technologies) according to the 2^−ΔΔ^*^C^*^t^ method [80].

### 4.11. Statistical Analyses

All data are expressed as the mean ± standard error of the mean (SEM). Differences between the control group and experimental groups (oxygen exposure and/or caffeine application) were analyzed using a two-way analysis of variance (ANOVA) with the factors hyperoxia exposure, caffeine, and their interaction. To determine differences between individual groups, each two-way ANOVA was followed by a Bonferroni post-hoc test. A two-sided *p*-value of <0.05 was considered significant. Results of the analyses were represented normalized as 100% value of control (24 h and 48 h, respectively). For the real-time PCR data, the 100% value of control represented the *C*_T_ values, while *C*_T_ was defined as the threshold cycle number of PCRs at which amplified product was first detected. For protein data (immunoblotting, ELISA, gelatin zymography), the 100% value of control represented the protein concentration or molarity (ELISA), intensity ratio of pixel size (immunoblotting), or inverted intensity of pixel size (gelatin zymography). All graphics and statistical analyses were performed using the GraphPad Prism 6.0 software for Windows (GraphPad Software, La Jolla, CA, USA).

## 5. Conclusions

In summary, our study reveals that single administration of caffeine in oxygen-induced brain injury leads to improved neuroprotective response. Exposure to high oxygen in the developmental brain of six-day-old rat pups resulted in increased levels of lipid peroxidation, enhanced generation of hydrogen peroxidase and heme oxygenase-1, decreased anti-oxidative response, up-regulated pro-inflammatory cytokines expression, changed balanced redox-sensitive reply, and promoted extracellular matrix degeneration and apoptotic cascade. All these consequences of hyperoxia can be reversed by caffeine treatment.

Our study suggests that caffeine is a drug that exerts neuroprotective effects on the developing brain due to its anti-oxidant, anti-inflammatory, and anti-apoptotic properties. Future research should focus on the investigation of caffeine for preconditioning and further enhance the knowledge about safety of caffeine use in the immature brain.

## Figures and Tables

**Table 1:** Sequences of oligonucleotides.

cDNA	Oligonucleotide Sequence 5′–3′	Accession No.
***HPRT***		
forward	GGAAAGAACGTCTTGATTGTTGAA	NM_012583.2
reverse	CCAACACTTCGAGAGGTCCTTTT	
probe	CTTTCCTTGGTCAAGCAGTACAGCCCC	
***Casp3***		
forward	ACAGTGGAACTGACGATGATATGG	NM_012922.2
reverse	AATAGTAACCGGGTGCGGTAGA	
probe	ATGCCAGAAGATACCAGTGG	
***GCLC***		
forward	GGAGGACAACATGAGGAAACG	NM_012815.2
reverse	GCTCTGGCAGTGTGAATCCA	
probe	GAGGCTACTTCTGTATTAGG	
***IFN-γ***		
forward	GCAAAAGGACGGTAACACGAA	NM_138880.2
reverse	ATGGCCTGGTTGTCTTTCAAGA	
probe	TCTCTTTCTACCTCAGACTC	
***IL-1β***		
forward	CTCCACCTCAATGGACAGAACA	NM_031512.2
reverse	CACAGGGATTTTGTCGTTGCT	
probe	CTCCATGAGCTTTGTACAAG	
***IL-12b***		
forward	TGCTGCTCCACAAGAAGGAA	NM_022611.1
reverse	TTGGTGCTTCACACTTCAGGAA	
probe	ATGGAATTTGGTCCACCGAG	
***IL-18***		
forward	CGGAGCATAAATGACCAAGTTCTC	NM_019165.1
reverse	TGGGATTCGTTGGCTGTTC	
probe	TTGACAAAAGAAACCCGCCTG	
***iNOS***		
forward	AGCTGTAGCACTGCATCAGAAATG	NM_012611.3
reverse	CAGTAATGGCCGACCTGATGT	
probe	CAGACACATACTTTACGCCAC	
***Keap1***		
forward	GATCGGCTGCACGGAACT	NM_057152.2
reverse	GCAGTGTGACAGGTTGAAGAACTC	
probe	CTCGGGAGTATATCTACATGC	
***NFκB1***		
forward	GACCCAAGGACATGGTGGTT	NM_001276711.1
reverse	TCATCCGTGCTTCCAGTGTTT	
probe	CTGGGAATACTTCACGTGAC	
***NFκB2***		
forward	GCCTAAACAGCGAGGCTTCA	NM_001008349.1
reverse	TCTTCCGGCCCTTCTCACT	
probe	TTTCGATATGGCTGTGAAGG	
***Nrf2***		
forward	ACTCCCAGGTTGCCCACAT	NM_031789.2
reverse	GCGACTCATGGTCATCTACAAATG	
probe	CTTTGAAGACTGTATGCAGC	
***SOD1***		
forward	CAGAAGGCAAGCGGTGAAC	NM_017050.1
reverse	CCCCATATTGATGGACATGGA	
probe	TACAGGATTAACTGAAGGCG	
***SOD2***		
forward	GACCTACGTGAACAATCTGAACGT	NM_017051.2
reverse	AGGCTGAAGAGCAACCTGAGTT	
probe	ACCGAGGAGAAGTACCACGA	
***SOD3***		
forward	GGAGAGTCCGGTGTCGACTTAG	NM_012880.1
reverse	CTCCATCCAGATCTCCAGGTCTT	
probe	CTGGTTGAGAAGATAGGCGA	
***TGF-β***		
forward	CCTGCAGAGATTCAAGTCAACTGT	NM_021578.2
reverse	GTCAGCAGCCGGTTACCAA	
probe	CAACAATTCCTGGCGTT	
***TIMP1***		
forward	CGGACCTGGTTATAAGGGCTAA	NM_053819.1
reverse	CGTCGAATCCTTTGAGCATCT	
probe	AGAAATCATCGAGACCACCT	
***TIMP2***		
forward	GGCAACCCCATCAAGAGGAT	NM_021989.2
reverse	GGGCCGTGTAGATAAATTCGAT	
probe	AGATGTTCAAAGGACCTGAC	
***tPa***		
forward	TCAGAAGAGGAGCTCGGTCCTA	NM_013151.2
reverse	TGGGACGTAGCCATGACTGAT	
probe	CAGAGATGAACAGACTCAGA	
***TNFα***		
forward	CCCCCAATCTGTGTCCTTCTAAC	NM_012675.2
reverse	CGTCTCGTGTGTTTCTGAGCAT	
probe	TAGAAAGGGAATTGTGGCTC	
